# Supplementary material for: The evolution of resource management in Taiwanese fisheries: coastal and offshore perspectives
Source: PeerJ. 2024 Nov 27;12:e18434. doi: 10.7717/peerj.18434 (PMC11608025; doi:10.7717/peerj.18434)
Supplement: Supplemental Information 3 [file peerj-12-18434-s003.docx]

| Family Name | Scientific name | Chinese Common name | Common name |
| --- | --- | --- | --- |
| Scombridae | *Thunnus orientalis* | 太平洋黑鮪 | Pacific Bluefin tuna |
| Scombridae | *Thunnus alalunga* | 長鰭鮪 | Albacore |
| Scombridae | *Katsuwonus pelamis* | 正鰹 | Skipjack tuna |
| Scombridae | *Thunnus albacares* | 黃鰭鮪 | Yellowfin tuna |
| Scombridae | *Thunnus obesus* | 大目鮪 | Bigeye tuna |
| Xiphiidae | *Xiphias gladius* | 旗魚 | Swordfish |
| Lamnidae | *Isurus oxyrinchus* | 尖吻鯖鯊 | Shortfin mako shark |
| Istiophoridae | *Istiompax indica* | 白皮旗魚 | Black marlin |
| Istiophoridae | Istiophorus platypterus | 平鰭旗魚 | Indo-pacific sailfish |
| Istiophoridae | *Kajikia audax* | 條紋四鰭旗魚 | Striped marlin |
| Istiophoridae | *Makaira mazara* | 黑皮旗魚 | Indo-Pacific blue marlin |
| Scombridae | *Euthynnus affinis* | 巴鰹 | Kawakawa |
| Coryphaenidae | *Coryphaena hippurus* | 鬼頭刀 | Common dolphinfish |
| Carcharhinidae | *Prionace glauca* | 水鯊 | Blue shark |
| Carcharhinidae | *-* | 黑鯊 | Requiem sharks  (blacktip shark) |
| Scomberesocidae | *Cololabis saira* | 秋刀魚 | Pacific saury |
| Molidae | *Mola mola* | 翻車魨 | Ocean sunfish |
| Exocoetidae | *Cypselurus unicolor* | 飛魚 | Whitefih flying fish |
| Scombridae | *Scomberomorus spp.* | 馬加鰆屬 | Spanish Mackerel |
| Scombridae | *Scomber australasicus* | 花腹鯖 | Blue mackerel |
| Rachycentridae | *Rachycentron canadum* | 海鱺 | Cobia |
| Scombridae | *Auxis spp.* | 圓花鰹 | Bullet tuna/Frigate tuna |
| Sphyraenidae | *Sphyraena barracuda* | 尖鮻 | Great barracuda |
| Gempylidae | *Ruvettus pretiosus* | 油魚 | Oilfish |
| Polynemidae | *Polydactylus sextarius* | 午仔魚 | Blackspot threadfin |
| Carcharhinidae | *Carcharhinus limbatus* | 黑鯊 | Blacktip shark |
| Triakidae | *Hemitriakis japonicus* | 鯊條 | Japanese topeshark |
| Trichiuridae | *Trichiurus lepturus* | 白帶魚 | Hairtail/Largehead hairtail |
| Carangidae | *Megalaspis cordyla* | 扁甲鰺 | Torpedo scad |
| Exocoetidae | Cheilopogon unicolor | 白鰭 | Limpid-wing flyingfish |
| Lutjanidae | *Lutjanus argentimaculatus* | 銀紋笛鯛 | Mangrove red snapper |
| Monacanthidae | *Aluterus monoceros* | 剝皮魚 | Unicorn leatherjacket filefish |
| Monacanthidae | *-* | 單棘魨科 | Filefishes (leatherjackets) |
| Carangidae | *Decapterus maruadsi* | 藍圓鰺 | Japanese scad |
| Carangidae | *Trachurus japonicus* | 真鰺 | Japanese jack mackerel |
| Dorosomatidae | *Sardinella sindensis* | 青鱗 | Sind sardinella |
| Mugilidae | *Mugil cephalus* | 烏魚 | Grey Mullet |
| Niphonidae | *Niphon spinosus* | 魚荒 | Ara |
| Carangidae | *Seriola dumerili* | 紅甘鰺 | Greater amberjack |
| Synodontidae | *Saurida elongata* | 狗母 | Slender lizardfish |
| Chirocentridae | *Chirocentrus dorab* | 西刀 | Dorab wolf herring |
| Sphyraenidae | *Sphyraena* | 魣 | Great barracuda |
| Muraenidae | *Enchelycore schismatorhynchus* | 海鰻 | White-margined moray |
| Caranginae | *Carangoides malabaricus* | 甘仔鰺 | Malabar trevally |
| Psettodidae | *Psettodes erumei* | 扁魚 | Indian halibut |
| Lutjanidae | *Lutjanus jocu* | 白鯛 | Dog snapper |
| Rajidae | *Amblyraja hyperborea* | 鰩 | Arctic skate |
| Epinephelidae | *Epinephelus malabaricus* | 鱠 | Malabar grouper |
| Priacanthidae | *Priacanthus macracanthus* | 紅目鰱 | Red Big-eye |
| Lutjanidae | *Lutjanus bohar* | 赤海 | Two-spot red snapper |
| Sciaenidae | *Pennahia macrocephalus* | 白口 | Big-head pennah croaker |
| Muraenidae | *-* | 鯙科 | Moray eels |
| Sciaenidae | *Pennahia argentata* | 白姑魚 | Silver croaker |
| Synodontidae | *Saurida* | 合齒魚科 | Lizardfishes; Bombay ducks |
| Epinephelidae | *Epinephelus coioides* | 點帶石斑 | Orange-spotted grouper |
| Latidae | *Psammoperca waigiensis* | 紅目鱸 | Sand Bass |
| Dasyatidae | *Bathytoshia lata* | 鬼魟 | Brown stingray |
| Epinephelidae | *Epinephelus lanceolatus* | 鞍帶石斑魚 | Giant grouper |
| Sciaenidae | *Nibea albiflora* | 黃姑魚 | Yellow drum |
| Centrolophidae | *Psenopsis anomala* | 刺鯧 | Japanese butterfish |
| Latidae | *Lates calcarifer* | 尖吻鱸 | Barramundi/ Giant seaperch |
| Nemiptidae | *Nemipterus virgatus* | 金線魚 | Golden thread |
| Sciaenidae | *Atrobucca nibe* | 黑口 | Blackmouth croaker |
| Carangidae | *Seriola quinqueradiata* | 鰤 | Japanese amberjack |
| Cynoglossidae | *Cynoglossus bilineatus* | 牛舌 | Fourlined tonguesole |
| Lethirinidae | *Lethrinus olivaceus* | 籠尖 | Longface emperor |
| Lutjanidae | *Lutjanus vitta* | 鬼魟 | Snappers / Brownstripe |
| Haemulidae | *-* | 鯙科 | Grunt fish |
| Sciaenidae | *Larimichthys crocea* | 黃花 | Large yellow croaker |
| Sparidae | *Dentex hypselosomus* | 黃背牙鯛 | Yellowback sea-bream |
| Lethrinidae | *Lethrinus miniatus* | 龍尖 | Trumpet emperor |
| Sciaenidae | *Larimichthys polyactis* | 黃花魚 | Yellow croaker |
| Sparidae | *Pagrus major* | 嘉臘 | Red seabream |
| Sparidae |  | 鯛科 | other seabream |
| Carangidae | *Alepes djedaba* | 吉打副葉鰺 | Shrimp scad |
| Triglidae | *Chelidonichthys ischyrus* | 角魚 | - |
| Macrouridae | *Coelorinchus formosanus* | 鱈魚 | Formosa grenadier |
| Sciaenidae | *Johnius distinctus* | 油口 | - |
| Sciaenidae | *Miichthys miiuy* | 鮸魚 | Mi-iuy croaker |
| Mobulidae | *Mobula alfredi* | 鲼 | Alfred manta |
| Dussumieriidae | *Etrumeus micropus* | 臭肉鰮 | Round herring |
| Menidae | *Mene maculata* | 皮刀 | Moonfish |
| Carangidae | *Decapterus kurroides* | 紅尾鰺 | Redtail scad |
| Sparidae | *Evynnis tumifrons* | 黃鯛 | Yellow seabream |
| Mullidae | *Parupeneus barberinus* | 秋姑 | Dash-and-dot goatfish |
| Latilidae | *Branchiostegus japonicus* | 馬頭 | Horsehead tilefish |
| Emmelichthyidae | *Erythrocles schlegelii* | 鰱魚 | Japanese rubyfish |
| Carangidae | *Trachurus japonicus* | 真鰺 | Japanese jack mackerel |
| Ariidae | *Arius maculatus* | 海鯰 | Sea catfish/Spotted sea catfish |
| Stromateidae | *Pampus echinogaster* | 鐮鯧 | Silver pomfret |
| Stromateidae | *Pampus argenteus* | 白鯧 | Silver pomfret |
| Sparidae | *Evynnis cardinalis* | 盤仔 | Threadfin porgy |
| Sparidae | *Acanthopagrus schlegelii* | 黑鯛 | Blackhead seabream |
| Sillaginidae | *Sillago asiatica* | 沙鮻 | Asian sillago |
| Spratelloididae | *Spratelloides gracilis* | 丁香 | Silver-stripe round herring |
| Lateolabracidae | *Lateolabrax japonicus* | 鱸魚 | Japanese seabass |
| Carangidae | *Parastromateus niger* | 黑鯧 | Black pomfret |
| Plecoglossidae | *Plecoglossus altivelis* | 香魚 | Ayu sweetfish |
| Dorosomatidae | *Nematalosa japonica* | 鰶 | Japanese gizzard shad |
| Scaridae | *-* | 鯙科 | parrotfish |
