# Supplementary material for: The evolution of resource management in Taiwanese fisheries: coastal and offshore perspectives
Source: PeerJ. 2024 Nov 27;12:e18434. doi: 10.7717/peerj.18434 (PMC11608025; doi:10.7717/peerj.18434)
Supplement: Supplemental Information 4 [file peerj-12-18434-s004.docx]

Table S1 The introduction for each species for this study

| Scenarios A | | Family Name | Scientific name | Common name | Habitat | TL | Descriptions/ Notes |
| --- | --- | --- | --- | --- | --- | --- | --- |
| NO. | Scenario 1 | Exclude Tuna | | | | | |
| 1 | 1 | Scombridae | *Thunnus orientalis* | Pacific Bluefin tuna | pelagic-oceanic | 4.05 | Highly migratory species |
| 2 | 2 |  | *Thunnus alalunga* | Albacore | pelagic-oceanic | 4.30 | Highly migratory species |
| 3 | 3 |  | *Katsuwonus pelamis* | Skipjack tuna | pelagic-oceanic | 4.40 | Highly migratory species |
| 4 | 4 |  | *Thunnus albacares* | Yellowfin tuna | pelagic-oceanic | 4.41 | Highly migratory species |
| 5 | 5 |  | *Thunnus obesus* | Bigeye tuna | pelagic-oceanic | 4.49 | Highly migratory species |
|  | Scenario 2 | Scenario 1 exclude oceanic migratory species | | | | | |
| 6 | 1 | Xiphiidae | *Xiphias gladius* | Swordfish | pelagic-oceanic | 4.51 | Highly migratory species |
| 7 | 2 | Lamnidae | *Isurus oxyrinchus* | Shortfin mako shark | pelagic-oceanic | 4.50 | Highly migratory species |
| 8 | 3 | Istiophoridae | *Istiompax indica* | Black marlin | pelagic-oceanic | 4.50 | Highly migratory species |
| 9 | 4 | Istiophoridae | Istiophorus platypterus | Indo-pacific sailfish | pelagic-oceanic | 4.50 | Highly migratory species |
| 10 | 5 | Istiophoridae | *Kajikia audax* | Striped marlin | pelagic-oceanic | 4.5 | Highly migratory species |
| 11 | 6 | Istiophoridae | *Makaira mazara* | Indo-Pacific blue marlin | pelagic-neritic | 4.5 | Highly migratory species |
| 12 | 7 | Scombridae | *Euthynnus affinis* | Kawakawa | pelagic-neritic | 4.485 | Highly migratory species |
| 13 | 8 | Coryphaenidae | *Coryphaena hippurus* | Common dolphinfish | pelagic-neritic | 4.4 | Highly migratory species |
| 14 | 9 | Carcharhinidae | *Prionace glauca* | Blue shark | pelagic-oceanic | 4.35 | Highly migratory species |
| 15 | 10 | Carcharhinidae | *-* | Requiem sharks (blacktip shark) | pelagic-oceanic | 4 | Highly migratory species |
| 16 | 11 | Scomberesocidae | *Cololabis saira* | Pacific saury | pelagic-oceanic | 3.71 | Highly migratory species |
| 17 | 12 | Molidae | *Mola mola* | Ocean sunfish | pelagic-oceanic | 3.50 | Highly migratory species |
| 18 | 13 | Exocoetidae | *Cypselurus unicolor* | Whitefih flying fish | pelagic-neritic | 3.25 | Highly migratory species |
|  | Scenario 3 | Scenario 2 exclude species with seasonal migration | | | | | |
| 19 | 1 | Scombridae | *Scomberomorus spp.* | Spanish Mackerel | pelagic-oceanic | 4.35 | Migratory fish |
| 20 | 2 | Scombridae | *Scomber australasicus* | Blue mackerel | pelagic-neritic | 4.23 | Migratory fish |
| 21 | 3 | Rachycentridae | *Rachycentron canadum* | Cobia | reef-associated | 4.26 | Migratory fish |
| 22 | 4 | Scombridae | *Auxis spp.* | Bullet tuna/Frigate tuna | pelagic-neritic | 4.24 | Migratory fish |
| 23 | 5 | Sphyraenidae | *Sphyraena barracuda* | Great barracuda | reef-associated | 4.19 | Migratory fish |
| 24 | 6 | Gempylidae | *Ruvettus pretiosus* | Oilfish | benthopelagic | 4.08 | Migratory fish |
| 25 | 7 | Polynemidae | *Polydactylus sextarius* | Blackspot threadfin | demersal | 4 | Migratory fish |
| 26 | 8 | Carcharhinidae | *Carcharhinus limbatus* | Blacktip shark | reef-associated | 4 | Migratory fish |
| 27 | 9 | Triakidae | *Hemitriakis japonicus* | Japanese topeshark | demersal | 4.00 | Migratory fish |
| 28 | 10 | Trichiuridae | *Trichiurus lepturus* | Hairtail/Largehead hairtail | benthopelagic | 3.99 | Migratory fish |
| 29 | 11 | Carangidae | *Megalaspis cordyla* | Torpedo scad | reef-associated | 3.91 | Migratory fish |
| 30 | 12 | Exocoetidae | Cheilopogon unicolor | Limpid-wing flyingfish | pelagic-neritic | 3.70 | Migratory fish |
| 31 | 13 | Lutjanidae | *Lutjanus argentimaculatus* | Mangrove red snapper | reef-associated | 3.58 | Migratory fish |
| 32 | 14 | Monacanthidae | *Aluterus monoceros* | Unicorn leatherjacket filefish | reef-associated | 3.49 | Migratory fish |
| 33 | 15 | Monacanthidae | *-* | Filefishes (leatherjackets) | reef-associated | 3.5 | Migratory fish |
| 34 | 16 | Carangidae | *Decapterus maruadsi* | Japanese scad | reef-associated | 3.4 | Migratory fish |
| 35 | 17 | Carangidae | *Trachurus japonicus* | Japanese jack mackerel | pelagic-neritic | 3.4 | Migratory fish |
| 36 | 18 | Dorosomatidae | *Sardinella sindensis* | Sind sardinella | pelagic-neritic | 2.9 | Migratory fish |
| 37 | 19 | Mugilidae | *Mugil cephalus* | Grey Mullet | benthopelagic | 2.5 | Migratory fish |
|  | Scenario 3 (the Indigenous Species around water of Taiwan) | | | | | | |
| 38 | 1 | Niphonidae | *Niphon spinosus* | Ara | reef-associated | 4.5 | Indigenous species |
| 39 | 2 | Carangidae | *Seriola dumerili* | Greater amberjack | reef-associated | 4.5 | Indigenous species |
| 40 | 3 | Synodontidae | *Saurida elongata* | Slender lizardfish | demersal | 4.5 | Indigenous species |
| 41 | 4 | Chirocentridae | *Chirocentrus dorab* | Dorab wolf herring | reef-associated | 4.4 | Indigenous species |
| 42 | 5 | Sphyraenidae | *Sphyraena* | Great barracuda | reef-associated | 4.4 | Indigenous species |
| 43 | 6 | Muraenidae | *Enchelycore schismatorhynchus* | White-margined moray | reef-associated | 4.38 | Indigenous species |
| 44 | 7 | Caranginae | *Carangoides malabaricus* | Malabar trevally | reef-associated | 4.36 | Indigenous species |
| 45 | 8 | Psettodidae | *Psettodes erumei* | Indian halibut | demersal | 4.35 | Indigenous species |
| 46 | 9 | Lutjanidae | *Lutjanus jocu* | Dog snapper | reef-associated | 4.35 | Indigenous species |
| 47 | 10 | Rajidae | *Amblyraja hyperborea* | Arctic skate | bathydemersal | 4.3 | Indigenous species |
| 48 | 11 | Epinephelidae | *Epinephelus malabaricus* | Malabar grouper | reef-associated | 4.2 | Indigenous species |
| 49 | 12 | Priacanthidae | *Priacanthus macracanthus* | Red Big-eye | reef-associated | 4.11 | Indigenous species |
| 50 | 13 | Lutjanidae | *Lutjanus bohar* | Two-spot red snapper | reef-associated | 4.1 | Indigenous species |
| 51 | 14 | Sciaenidae | *Pennahia macrocephalus* | Big-head pennah croaker | demersal | 4.08 | Indigenous species |
| 52 | 15 | Muraenidae | *-* | Moray eels | reef-associated | 4.1 | Indigenous species |
| 53 | 16 | Sciaenidae | *Pennahia argentata* | Silver croaker | benthopelagic | 4.06 | Indigenous species |
| 54 | 17 | Synodontidae | *Saurida* | Lizardfishes; Bombay ducks | reef-associated | 4 | Indigenous species |
| 55 | 18 | Epinephelidae | *Epinephelus coioides* | Orange-spotted grouper | reef-associated | 4 | Indigenous species |
| 56 | 19 | Latidae | *Psammoperca waigiensis* | Sand Bass | reef-associated | 4 | Indigenous species |
| 57 | 20 | Dasyatidae | *Bathytoshia lata* | Brown stingray | demersal | 4 | Indigenous species |
| 58 | 21 | Epinephelidae | *Epinephelus lanceolatus* | Giant grouper | reef-associated | 4 | Indigenous species |
| 59 | 22 | Sciaenidae | *Nibea albiflora* | Yellow drum | benthopelagic | 4 | Indigenous species |
| 60 | 23 | Centrolophidae | *Psenopsis anomala* | Japanese butterfish | benthopelagic | 4 | Indigenous species |
| 61 | 24 | Latidae | *Lates calcarifer* | Barramundi/ Giant seaperch | demersal | 4 | Indigenous species |
| 62 | 25 | Nemiptidae | *Nemipterus virgatus* | Golden thread | demersal | 3.99 | Indigenous species |
| 63 | 26 | Sciaenidae | *Atrobucca nibe* | Blackmouth croaker | demersal | 3.96 | Indigenous species |
| 64 | 27 | Carangidae | *Seriola quinqueradiata* | Japanese amberjack | demersal | 3.96 | Indigenous species |
| 65 | 28 | Cynoglossidae | *Cynoglossus bilineatus* | Fourlined tonguesole | demersal | 3.89 | Indigenous species |
| 66 | 29 | Lethirinidae | *Lethrinus olivaceus* | Longface emperor | reef-associated | 3.85 | Indigenous species |
| 67 | 30 | Lutjanidae | *Lutjanus vitta* | Snappers / Brownstripe | reef-associated | 3.8 | Indigenous species |
| 68 | 31 | Haemulidae | *-* | Grunt fish | reef-associated | 3.8 | Indigenous species |
| 69 | 32 | Sciaenidae | *Larimichthys crocea* | Large yellow croaker | benthopelagic | 3.7 | Indigenous species |
| 70 | 33 | Sparidae | *Dentex hypselosomus* | Yellowback sea-bream | demersal | 3.7 | Indigenous species |
| 71 | 34 | Lethrinidae | *Lethrinus miniatus* | Trumpet emperor | reef-associated | 3.7 | Indigenous species |
| 72 | 35 | Sciaenidae | *Larimichthys polyactis* | Yellow croaker | benthopelagic | 3.7 | Indigenous species |
| 73 | 36 | Sparidae | *Pagrus major* | Red seabream | demersal | 3.7 | Indigenous species |
| 74 | 37 | Sparidae |  | other seabream | demersal | 3.7 | Indigenous species |
| 75 | 38 | Carangidae | *Alepes djedaba* | Shrimp scad | reef-associated | 3.58 | Indigenous species |
| 76 | 39 | Triglidae | *Chelidonichthys ischyrus* | - | demersal | 3.5 | Indigenous species |
| 77 | 40 | Macrouridae | *Coelorinchus formosanus* | Formosa grenadier | benthopelagic | 3.50 | Indigenous species |
| 78 | 41 | Sciaenidae | *Johnius distinctus* | - | benthopelagic | 3.5 | Indigenous species |
| 79 | 42 | Sciaenidae | *Miichthys miiuy* | Mi-iuy croaker | demersal | 3.5 | Indigenous species |
| 80 | 43 | Mobulidae | *Mobula alfredi* | Alfred manta | benthopelagic | 3.5 | Indigenous species |
| 81 | 44 | Dussumieriidae | *Etrumeus micropus* | Round herring | pelagic-neritic | 3.5 | Indigenous species |
| 82 | 45 | Menidae | *Mene maculata* | Moonfish | reef-associated | 3.45 | Indigenous species |
| 83 | 46 | Carangidae | *Decapterus kurroides* | Redtail scad | reef-associated | 3.4 | Indigenous species |
| 84 | 47 | Sparidae | *Evynnis tumifrons* | Yellow seabream | demersal | 3.4 | Indigenous species |
| 85 | 48 | Mullidae | *Parupeneus barberinus* | Dash-and-dot goatfish | reef-associated | 3.4 | Indigenous species |
| 86 | 49 | Latilidae | *Branchiostegus japonicus* | Horsehead tilefish | demersal | 3.4 | Indigenous species |
| 87 | 50 | Emmelichthyidae | *Erythrocles schlegelii* | Japanese rubyfish | demersal | 3.4 | Indigenous species |
| 88 | 51 | Carangidae | *Trachurus japonicus* | Japanese jack mackerel | pelagic-neritic | 3.4 | Indigenous species |
| 89 | 52 | Ariidae | *Arius maculatus* | Sea catfish/Spotted sea catfish | demersal | 3.36 | Indigenous species |
| 90 | 53 | Stromateidae | *Pampus echinogaster* | Silver pomfret | benthopelagic | 3.3 | Indigenous species |
| 91 | 54 | Stromateidae | *Pampus argenteus* | Silver pomfret | benthopelagic | 3.3 | Indigenous species |
| 92 | 55 | Sparidae | *Evynnis cardinalis* | Threadfin porgy | reef-associated | 3.3 | Indigenous species |
| 93 | 56 | Sparidae | *Acanthopagrus schlegelii* | Blackhead seabream | demersal | 3.24 | Indigenous species |
| 94 | 57 | Sillaginidae | *Sillago asiatica* | Asian sillago | demersal | 3.200 | Indigenous species |
| 95 | 58 | Spratelloididae | *Spratelloides gracilis* | Silver-stripe round herring | pelagic-neritic | 3.1 | Indigenous species |
| 96 | 59 | Lateolabracidae | *Lateolabrax japonicus* | Japanese seabass | reef-associated | 3.1 | Indigenous species |
| 97 | 60 | Carangidae | *Parastromateus niger* | Black pomfret | reef-associated | 2.9 | Indigenous species |
| 98 | 61 | Plecoglossidae | *Plecoglossus altivelis* | Ayu sweetfish | demersal | 2.8 | Indigenous species |
| 99 | 62 | Dorosomatidae | *Nematalosa japonica* | Japanese gizzard shad | benthopelagic | 2.4 | Indigenous species |
| 100 | 63 | Scaridae | *-* | Parrotfish | reef-associated | 2 | Indigenous species |
